# Deconvoluting the Optical Response of Biocompatible Photonic Pigments

**DOI:** 10.1002/ange.202206562

**Published:** 2022-07-13

**Authors:** Zhen Wang, Chun Lam Clement Chan, Johannes S. Haataja, Lukas Schertel, Ruiting Li, Gea T. van de Kerkhof, Oren A. Scherman, Richard M. Parker, Silvia Vignolini

**Affiliations:** ^1^ Melville Laboratory for Polymer Synthesis Yusuf Hamied Department of Chemistry University of Cambridge Lensfield Road Cambridge CB2 1EW UK; ^2^ Department of Physics University of Fribourg Chemin du Musée 3 1700 Fribourg Switzerland

**Keywords:** Block Copolymers, Confinement, Inverse Photonic Glasses, Self-Assembly, Structural Color

## Abstract

To unlock the widespread use of block copolymers as photonic pigments, there is an urgent need to consider their environmental impact (cf. microplastic pollution). Here we show how an inverse photonic glass architecture can enable the use of biocompatible bottlebrush block copolymers (BBCPs), which otherwise lack the refractive index contrast needed for a strong photonic response. A library of photonic pigments is produced from poly(norbornene‐*graft*‐polycaprolactone)‐*block*‐poly(norbornene‐*graft*‐polyethylene glycol), with the color tuned via either the BBCP molecular weight or the processing temperature upon microparticle fabrication. The structure–optic relationship between the 3D porous morphology of the microparticles and their complex optical response is revealed by both an analytical scattering model and 3D finite‐difference time domain (FDTD) simulations. Combined, this allows for strategies to enhance the color purity to be proposed and realized with our biocompatible BBCP system.

## Graphical abstract

Inverse photonic glass microparticles, with colors across the visible spectrum, were prepared from a biocompatible bottlebrush block copolymer. The structure–optic relationship was investigated by i) a 3D phase‐field foam structural model and FDTD optical simulation, and ii) an analytical model of an assembly of Mie scatterers. From this, a strategy to improve the color purity was proposed and realized.

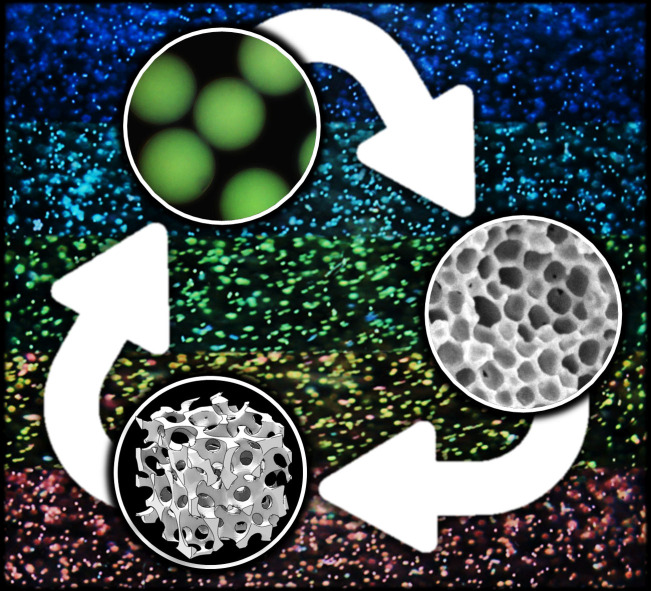

## Introduction

Bottlebrush block copolymers (BBCPs) have drawn increasing attention for their ability to self‐assemble into well‐ordered nanostructures with a periodicity on the order of the wavelengths of visible light, resulting in vibrant structural color.[^1^, ^2^, ^3^, ^4^, ^5^] However, as their use as photonic materials grows from laboratory‐scale demonstrations to coatings and effect pigments, there is a growing need to replace traditional BBCP systems with more sustainable and biocompatible alternatives.[^6^, ^7^] Unfortunately, vibrant coloration typically requires a large refractive index contrast between the BBCP domains (Δ*n*>0.1), which is challenging to achieve with common biocompatible polymers, such as: polycaprolactone (*n*=1.46–1.48),[^8^, ^9^] polylactide (*n*=1.44–1.48),[^10^, ^11^] polyethylene glycol (*n*=1.46),^[12]^ or poly(2‐hydroxyethyl methacrylate) (*n*=1.51).^[13]^ As such, substituting for biocompatible polymers imposes stringent restrictions on the design of the photonic structure.

To circumvent this limitation, one can consider inverse photonic glass architectures,[^14^, ^15^, ^16^, ^17^] where the intensity of the color is instead determined by the refractive index contrast between the cavities (typically air or solvent), and the surrounding matrix. Although such structures have been produced through confined self‐assembly of BBCPs,[^18^, ^19^] they in general suffer from lower overall intensity and poor color purity at longer wavelengths.^[20]^ However, such limitations are mainly due to the lack of a complete parameter space investigation of such complex 3D architectures. Indeed, the theoretical studies reported so far to probe the structure‐optic properties of inverse photonic glass architectures either relied on Monte‐Carlo methods or finite‐difference time domain (FDTD) simulations of simplified 2D geometries.[^20^, ^21^, ^22^] Therefore, the ability to model an experimentally relevant 3D structure that can be used for a full FDTD calculation will enable the investigation of a wide structural parameter space, avoiding extensive and tedious sample preparation and characterization.

In this work, a series of inverse photonic glass microparticles were prepared from a biocompatible BBCP, which contains sidechains of both polycaprolactone (PCL) and polyethylene glycol (PEG). By tuning either synthetic parameters (i.e. BBCP molecular weight) or fabrication conditions (i.e. evaporation temperature), the reflected color was precisely tuned across the entire visible spectrum, allowing for trends in the structure‐optic relationships to be identified. To investigate the optical response, the 3D porous structure was first modelled using a 3D phase‐field foam simulation and then the optical response calculated using a FDTD approach. This enabled us to deconvolute the origin of the photonic response of such systems in terms of structural parameters (such as wall filling fraction and correlation length) and consequently design the spectral response of the microparticles. Finally, the origin of the different spectral peaks was revealed using an analytical model for a Mie scatterer and an assembly of scatterers, which when combined with insights from the 3D FDTD simulation enabled a strategy to improve the optical response (in terms of vibrancy and color purity) to be proposed and subsequently realized.

## Results and Discussion

**Figure 1:**
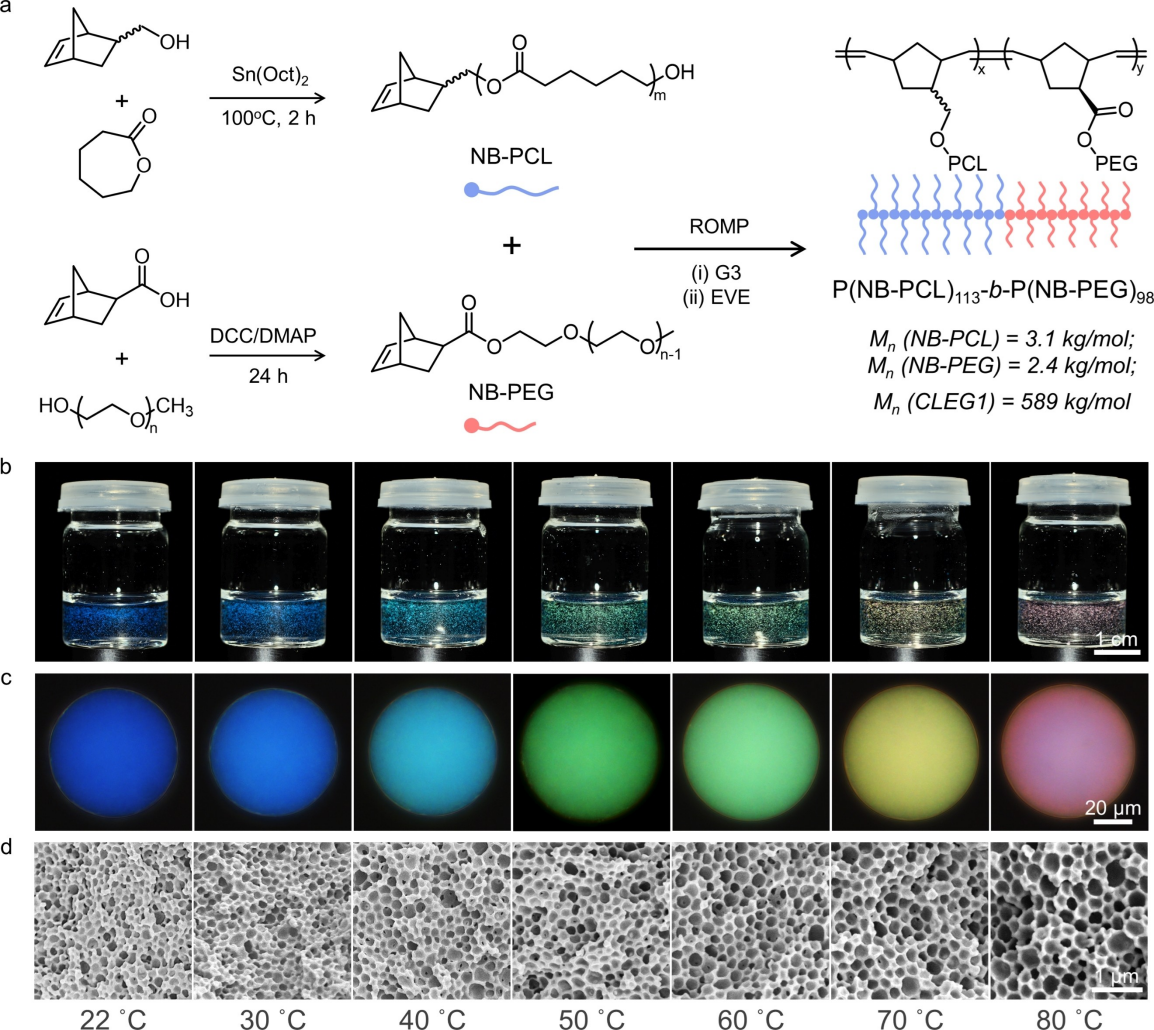
a) Schematic of the synthetic route for the biocompatible BBCP, including preparation of the two macromonomers, NB‐PCL and NB‐PEG, and their copolymerization via ring‐opening metathesis polymerization (ROMP) to form P(NB‐PCL)‐*b*‐P(NB‐PEG); the number average molecular weights (M_n_) are also included. b) Photographs recorded under direct illumination of aqueous dispersions of photonic BBCP pigments, formed from the controlled evaporation of monodisperse toluene/water microdroplets at different temperatures (*T*=22–80 °C). c) Corresponding micrographs of individual BBCP microspheres for each temperature, showing a rainbow of colors. d) Cross‐sectional SEM images reveal the internal porous nanoarchitecture of this series of photonic pigments.

The polymers PCL and PEG have been reported to be both biocompatible and degradable,[^23^, ^24^, ^25^, ^26^] and as such were selected as the sidechains, while norbornene was used to form the backbone of the BBCP (Figure 1a). The PCL macromonomer (NB‐PCL) was obtained by ring‐opening polymerization (ROP) of caprolactone (CL), initiated by 5‐norbornene‐2‐methanol (synthetic methods in Section S1, Supporting Information). The polymerization kinetics were optimized by gel permeation chromatography (GPC), with a polymerization time of 2 h selected to ensure good conversion while minimizing polymer dispersity (Figure S1 and Table S1, Supporting Information). The average degree of polymerization (DP) and number average molecular weight (M_n_) were determined by ^1^H‐NMR spectroscopy to be 26.5 and 3.1 kg mol^−1^, respectively (Figure S2, Supporting Information). The PEG macromonomer (NB‐PEG) was synthesized via an esterification of a commercially available polyethylene glycol methyl ether, with an average DP of 49.6 and a M_n_ of 2.4 kg mol^−1^ (Section S1 and Figure S3, Supporting Information).

A grafting‐through method was used to prepare poly(norbornene‐*graft*‐polycaprolactone)‐*block*‐poly(norbornene‐*graft*‐polyethylene glycol), P(NB‐PCL)‐*b*‐P(NB‐PEG), from the NB‐PCL and NB‐PEG macromonomers, via ring‐opening metathesis polymerization (ROMP) using third generation Grubbs catalyst (G3), see Section S1, Supporting Information. To achieve both a narrow polymer dispersity and a well‐defined block ratio, precise control over the amount of initiator added was achieved using a syringe pump (Figure S4, Supporting Information). This also allowed for the polymerization kinetics of the two macromonomers to be investigated by GPC. For NB‐PCL, efficient polymerization occurred in only 3 min (Figure S5, Supporting Information), while for NB‐PEG a conversion of 96 % required 20 min, increasing to 100 % after 30 min (Figure S6, Supporting Information). Using these optimized reaction conditions, a family of BBCPs with precise control over the DP and M_n_ was produced (Table S2, Supporting Information). From this family, **CLEG1** was selected for the following studies, with an absolute M_n_ of 589 kg mol^−1^ and a composition of P(NB‐PCL)_113_‐*b*‐P(NB‐PEG)_98_ (Figure S7 and S8, Supporting Information).

To produce photonic pigments, a solution of **CLEG1** in toluene (30 mg mL^−1^) was emulsified in water using a flow‐focusing microfluidic device (see Section S1 and Figure S9, Supporting Information). The resultant microdroplets were evaporated, with the complete loss of toluene resulting in a dispersion of structurally colored microparticles (Figure 1b). As shown in Figure 1c, highly uniform color can be observed across the entire microparticle when imaged in epi‐illumination. By varying the evaporation temperature, the color of the microparticles could be tuned across the visible spectrum, ranging from blue (*T*=22 °C) to red (*T*=80 °C), with micro‐spectroscopy confirming a linear relationship between the wavelength of the main reflection peak and the evaporation temperature (Figure S10). To confirm the internal structure of these “photonic pigments”, the microparticles were freeze‐dried and fractured, with the cross‐sections then imaged using a scanning electron microscope (SEM). As shown in Figure 1d, this analysis revealed a typical inverse photonic glass architecture.^[18]^ Furthermore, cryo‐SEM imaging of a sublimated sample verified that this porous structure exists in the microparticles dispersed in aqueous solution, rather than formed by this freeze drying process (Figure S11).

The origin of the porous architecture has been previously attributed to the formation of nanoscale water droplets within the larger toluene microdroplet,[^18^, ^27^] but how this results in the narrow distribution of pore sizes required for a photonic response is not well understood. Here, we confirm that **CLEG1** can act as a “giant surfactant” (interfacial tension γ=+16.5 mN m^−1^, see Figure S12 and Section S7, Supporting Information) and that in the presence of water it can promote the formation of micelles (Z‐average size of 284 nm after 5 min at 22 °C, see Figure S13, Supporting Information). Importantly, over time these micelles can swell while maintaining a narrow size distribution (PDI=0.1–0.5, see Figure S13, Supporting Information), with the degree of swelling dependent on the temperature. As explained further in Section S7 of the Supporting Information, this supports the mechanism of micelles first forming upon emulsification of the BBCP toluene solution in water, and then geometrically packing upon subsequent evaporation of these toluene droplets to yield the uniform porous structures observed in Figure 1d, with the BBCP shells of the micelles fusing upon final drying to form the extended polymer matrix.

The optical response and the structural properties of the microparticles were investigated using bright‐field micro‐spectroscopy and cross‐sectional SEM imaging, respectively. It is clear that the spectrum of most microparticles exhibit two peaks (Figure S14a–d, Supporting Information), which seemingly agrees with previous examples reported in the literature.^[18]^ The position of the higher wavelength peak in the spectrum (taken to be the “structural peak”, S_p_[^7^, ^18^, ^28^]) was found to redshift when the evaporation temperature was increased (Figure S14e, Supporting Information). In accordance with Bragg's law, the position of the redshifted peak scales linearly with the correlation length (*ξ*), which is proportional to the average center‐to‐center distance between each pore [cf. Eq. (S3) and Figure S15a, b, Supporting Information]. Furthermore, structural analysis (as summarized in Section S3, Supporting Information), reveals that the increase in correlation length results primarily from an increase in the average pore radius (Figure S15–S18, Supporting Information), with the wall thickness not significantly changing (Figure S19, Supporting Information). Accordingly, the filling fraction (*ff*, defined as the ratio of the wall relative to the total volume), decreases as this “structural peak” redshifts (Figure S15d, Supporting Information). For example, a blue microsphere has a correlation length of 83 nm and a filling fraction of 53 % while the corresponding values for a red microsphere are 125 nm and 31 %, respectively. Beyond the average diameter of the pores, their size distribution (*w_r_*, defined as the full width at half maximum, see Figure S16, S17 and Section 3 in Supporting Information), also plays a crucial role in the degree of correlation of the porous structure, which ultimately affects the intensity of the “structural peak”. It was found that upon increasing the evaporation temperature, *w_r_* increased (Figure S17b, Supporting Information), with a corresponding increase in the width of the structural peak and a reduction in its reflection intensity (Figure S10a, Supporting Information).

To understand how structural characteristics (correlation length, filling fraction etc) individually or collectively influence the visual appearance of the photonic pigments (peak wavelength, intensity etc), the correlated porous structure was simulated. In contrast to previous 2D methods in the literature,[^20^, ^28^] here we developed a new approach that allowed for the porous morphology of microparticles to be approximated using phase‐field models for a 3D foam structure.^[29]^ This allowed for artificial photonic glasses with the desired structural parameters to be “synthesized in silico”, which could then be used in FDTD simulations to predict their optical properties (see Section 4 in Supporting Information for details). Using this approach, we were able to mimic experimentally realistic structures for different combinations of correlation length (*ξ*_sim_=100–140 nm) and filling fraction (*ff*_sim_=25–55 %).

**Figure 2:**
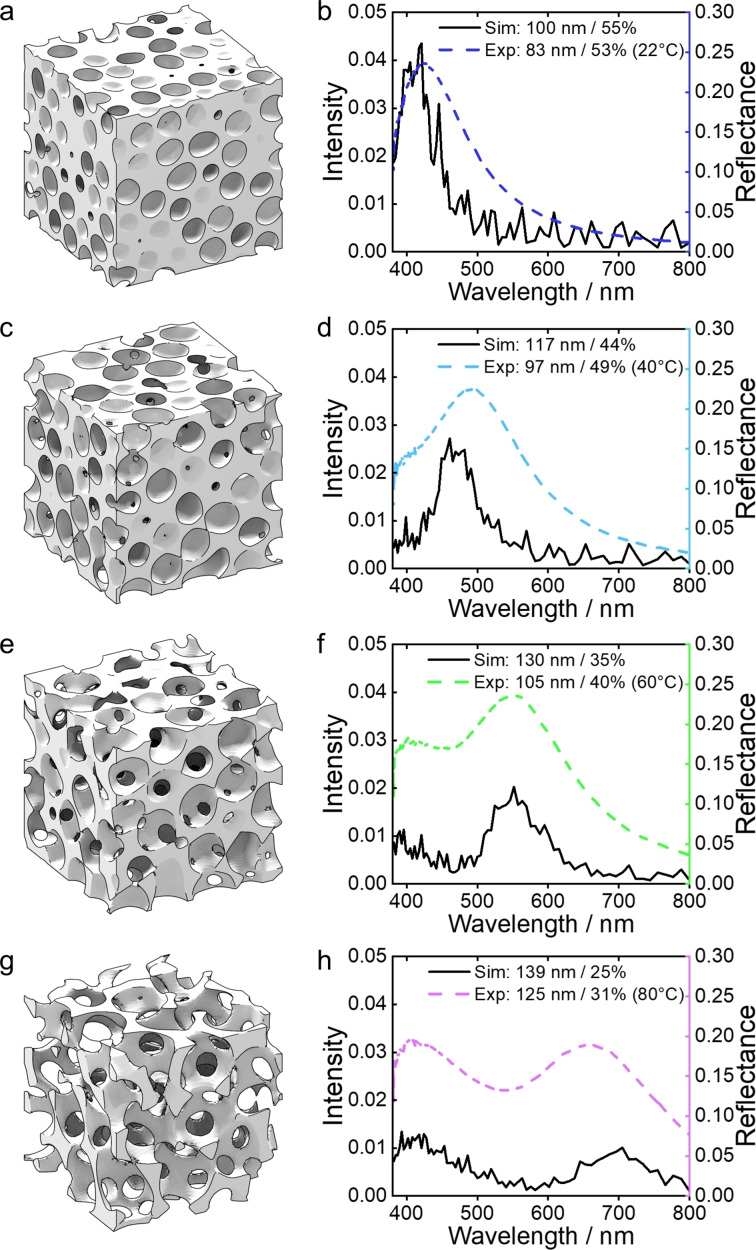
Finite difference time domain (FDTD) simulations on in silico synthesized foam structures: a), c), e), g) Two‐phase 3D models representing the photonic structures with different correlation lengths (*ξ*_sim_) and filling fraction (*ff*_sim_). The structural parameters of each structure, denoted *ξ*_sim_ / *ff*_sim_ are: a) 100 nm/55 %, c) 117 nm/44 %, e) 130 nm/35 %, g) 139 nm/25 %; b), d), f), h) Simulated optical response (left axis; black solid lines) for these in silico synthesized structures and experimental spectra (right axis; colored dash lines) measured for photonic pigments prepared at 22, 40, 60 and 80 °C (from top to bottom).

The FDTD simulations showed that for a fixed *ff*_sim_ of 55 %, increasing *ξ*_sim_ over this range leads to a linear increase in the wavelength of the structural peak (Figure S20, Supporting Information), resulting in a redshift from *λ*≈420 nm to 560 nm, in agreement with Bragg's law. In contrast, for a fixed *ξ*_sim_ of 100 nm, increasing *ff*_sim_ from 25 % to 55 % results in a blueshift in peak position combined with a significant increase in peak amplitude, suggesting that filling fraction is potentially determinant for the reflected intensity as well as tuning the color (Figure S21, Supporting Information). Given that the structural characterization methods either over‐ or under‐evaluate the structural parameters, as explained in Figure S11, the efficacy of the model was also assessed by correlating simulated spectra against that of the experimental temperature series. As shown in Figure 2, the simulated spectral peaks match well with the experimental data in terms of both the number of peaks and their relative intensities and positions. Furthermore, the simulated structural parameters are in good agreement with those derived from SEM, with the temperature trends reproduced. The differences (notably intensity) can be attributed to the discrepancy in the system sizes, as the simulated cube (5 μm×5 μm×5 μm) is much smaller than the experimental microparticle (i.e. a sphere with diameter ≈70 μm), and to the fact that idealized foam model may not be a perfect match to the experimental structures. Although increasing the simulated volume size by a factor of 8 (i.e. doubling the simulation box length *N*) results in a higher reflectance intensity that better matches experimental observations (Figure S22, Supporting Information), the cubical memory and computation time dependence on *N* renders the endeavor to match the experimental volume infeasible.

Importantly, as *ξ*_sim_ increases and *ff*_sim_ decreases, a second peak at low wavelength moves further into the visible region, consistent with what was observed for samples dried at higher temperatures. This second peak plays an increasing role in the overall visual appearance, which is most striking for the red sample (Figure 2h), where the reflection at 420 nm has a higher intensity than from the “structural peak” at 700 nm, resulting in a loss of color purity. This issue of reduced color purity at larger wavelengths is not unique to our system and is well‐known for both natural and fabricated photonic glasses.[^20^, ^30^]

To understand the origin of this secondary peak, an analytical description of scattering was used to deconvolute the contribution of different effects, allowing us to interpret which morphological parameters are most relevant for tuning the system towards greater color purity. The structural color of (inverse) photonic glasses depends on multiple parameters, such as system size (often referred to as thickness, *L*), the degree of correlation between the scattering elements (structure factor, *S*(*θ*)) and the shape and size of the scattering elements (represented by the form factor, *F*(*θ*)).[^20^, ^28^] In a system with multiple scattering elements, the ratio of the system size and the so‐called transport mean free path l* (length scale on which the direction of the light is randomized) determines the regime of light transport. If *L*≫l*, it is multiple scattering (i.e. white diffusive). For *L*≪l* it is in the single scattering regime, while *L*≈l* it is in a semitransparent scattering regime. Interestingly, it was found that isotropic structural color is most pure in this transition regime,^[28]^ partly explained by the fact that multiple scattering is largely suppressed while coherent scattering effects are still present. For a low refractive index system surrounded by a liquid medium, the mean free path is expected to be on the order of tens of microns,^[20]^ which is the size regime of our inverse photonic glass microparticles.

**Figure 3:**
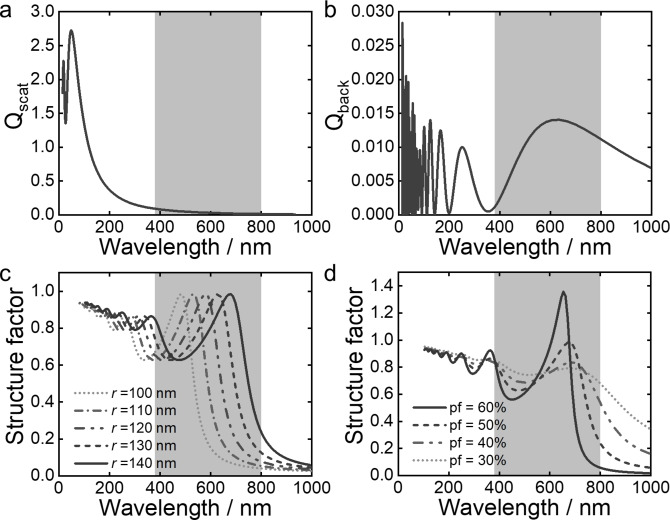
a) Total scattering cross section of a Mie sphere (*r*=140 nm, *n*_pore_=1.33, *n*_matrix_=1.5). b) Backscattering cross section of the same Mie sphere. c) Percus–Yevick structure factor for a packing fraction (pf) of 50 %, with *n*_pore_=1.33, *n*_matrix_=1.5 and varying radius. d) Structure factor for *r*=140 nm and varying pf. The grey band indicates the wavelength range in Figure 2.

For resonant scattering systems with a structural correlation, such as in this case, it is not trivial to determine if the contribution from single scattering or the overall multiple scattering from the form factor (as defined in the Supporting Information) dominates the spectral response throughout the visible wavelength range.^[31]^ Therefore, these two contributions were plotted separately to discriminate their contribution. Figure 3a shows the total differential scattering cross section *Q*_scat_ (averaged over all angles) of a Mie sphere with the given properties of the experimental system (*r*=140 nm, *n*_pore_=1.33, *n*_matrix_=1.5) calculated with Mie theory.^[32]^ No resonant peak falls in the visible wavelength range (grey marked section) for this low refractive index regime. In our system one could assume that multiple scattering is very low, and the contributions from single scattering may likely dominate the optical response, which are better represented by the backscattering cross section *Q*_back_ (differential scattering cross section only in backscattering direction), as plotted in Figure 3b. Here the first Mie resonance falls in the visible region. Note however, that the peak is much wider than the experimentally observed spectra and the relative intensity is two orders of magnitude lower than for the total cross section. Furthermore, both the experimentally observed polydispersity and non‐spherical shape of the scatters might further lower the importance of this contribution.

We thus plot the structural contribution in Figure 3c (for various radii) and Figure 3d (for various co‐packing fractions, which equate to the degree of correlation or the “correlation strength”). Here we use the hard‐sphere Percus–Yevick structure factor approximation^[33]^ accounting for the effective refractive index of the system according to the Garnett effective index.[^34^, ^35^] The peak generated by the structural contribution in the visible region (first order structural peak) corresponds well to the position of the peaks observed in both experimental spectra and the FDTD simulations. The shift of the first order structural peak toward larger wavelength as well as the appearance of a second peak in the blue region (second order structural peak) of the spectrum is well explained in Figure 3c. This is in contrast to earlier publications,^[30]^ where a low wavelength peak (hindering pure red structural color) was often associated with a Mie resonance. As a consequence this peak cannot be simply shifted outside the visible spectrum by lowering the scatterer size while keeping the correlation length the same, as suggested previously for 2D systems.^[20]^ An alternative design strategy is revealed by Figure 3d, which shows the dependence of the peak height on the correlation strength (packing fraction in the case of hard spheres). This plot suggests that a more ordered (i.e. stronger) packing could lead to an enhanced first order structural peak without significant enhancement to the second order structural peak, leading to a purer red color. This enhancement in peak ratio is also validated by FDTD simulations, where for *ξ*_sim_=140 nm an increase in *ff*_sim_ from 25 % to 55 % leads to a three‐fold increase in the intensity of the first order structural peak alone, albeit with an associated blueshift (Figure S23, Supporting Information).

The insights from the analytical model, combined with the FDTD simulation, suggest improving correlation strength as a design strategy to optimize for red colors that are more pure. In the experimental system, the filling fraction (*ff*) affects the correlation strength analogously to the packing fraction in the hard spheres model. Furthermore, the experimental pore size distribution (*w_r_*) is an important metric for the packing quality, which is not considered in the monodisperse hard spheres model. Intuitively, incorporating NB‐PCL macromonomer into the BBCP formulation used to prepare the photonic pigment should result in microparticles with thicker walls, while maintaining the pore size and distribution, resulting in an increased filling fraction and a small increase in correlation length (and thus a redshift of the color). Experimentally it was found that when NB‐PCL was included in moderate amounts (e.g. 40 wt % relative to **CLEG1**), the resultant microparticles showed increased reflectance (approx. 180 %), but their color was also blueshifted (Δ*λ*≈−35 nm). This unexpected blueshift can be explained by reduced water uptake within the toluene droplet upon increasing the amount of hydrophobic NB‐PCL, resulting in a decreased average pore size and correlation length, despite the increase in wall thickness (Figure S24, Supporting Information). This correlation between peak intensity and the filling fraction is also observed in the FDTD simulation (Figure S25, Supporting Information). Notably, higher NB‐PCL loadings (>80 wt %) resulted in loss of the well‐defined porous structure, leading to only a weak blue color.

In order to keep the position of the reflected peak constant while increasing the filling fraction, the evaporation temperature was thus increased in conjunction with NB‐PCL blending. With an increase in temperature, the peak redshifts to higher wavelengths, but also broadens due to the increased pore size distribution. Conversely, introducing NB‐PCL increases the filling fraction, leading to a narrower pore size distribution, resulting in a blueshift and a higher first order structural peak. As such the combined effect of these two parameter controls should allow for a fine tuning of the hue, intensity, and color purity of the photonic pigments.

**Figure 4:**
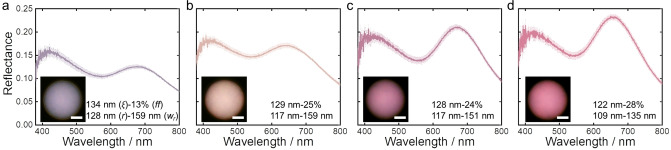
Reflectance spectra and microscope images (inset) for CLEG4 microspheres prepared in different conditions to enhance the contribution of the first structural peak towards a purer red color. The evaporation temperature and macromonomer loading are: a) 60 °C with no NB‐PCL; b) 60 °C with 20 % NB‐PCL; c) 65 °C with 25 % NB‐PCL; d) 65 °C with 30 % NB‐PCL. The correlation length (*ξ*), filling fraction (*ff*), pore radius (*r*), and pore size distribution (*w_r_*) determined from structural analysis are also stated. Scale bar for insets is 20 μm.

To ensure that in the absence of any NB‐PCL the first order structural peak is within the red spectral region, a second BBCP with much larger molecular weight (**CLEG4**: P(NB‐PCL)_206_‐*b*‐P(NB‐PEG)_187_, with a M_n_ of 1095 kg mol^−1^) was employed (Figure S26 and Table S2, Supporting Information). Under the microscope, a microparticle prepared at 60 °C with **CLEG4** has a desaturated purple appearance, attributed to a weak first order structural peak that allows the second order peak to dominate the color. This is a result of the weak correlation strength of these pores in terms of low *ff* (13 %) and high *w_r_* (159 nm), leading to a modest contribution of scattering to the main structural reflection peak (Figure 4a and S27a, Supporting Information). Simply increasing the temperature to 65 °C redshifts the peak wavelength and further weakens the correlation strength (*ff*=9 %, *w_r_* =171 nm), leading to an overall greater blue contribution in the purple microparticle (Figure S28). Conversely, blending in 20 wt % NB‐PCL and evaporating at 60 °C resulted in microparticles with a blueshifted, more intense structural peak that contributes more significantly to the final color, as indicated by a decreased intensity ratio between the positions at *λ*=420 and 640 nm (Figure 4b). Therefore, by increasing the temperature in combination with doping with NB‐PCL, the structural peak position can be maintained while improving the correlation strength, resulting in a magenta‐red microparticle (Figure 4c). As shown in Figure 4d, this can be optimized further to achieve a purer red color, attributed to the higher *ff* (28 %) and lower *w_r_* (135 nm) from SEM analysis. Beyond this, further increases in temperature and macromonomer loading did not result in any additional improvements in the color purity (Figure S29, Supporting Information). Importantly, the invariance of the position of the peak in the blue region, irrespective of filling fraction (Figure 4) strengthens our hypothesis that this peak arises from second order structural correlation rather than from a distinct Mie resonance, as previously proposed.^[20]^

Finally, given that it is known that the visual appearance of porous BBCP microparticles can be tuned by both the preparation conditions^[18]^ and the molecular weight of BBCP,^[27]^ it is useful to compare the efficacy of each method. As noted previously, a series of BBCPs with similar block ratios but increasing M_n_ were synthesized (**CLEG1**–**4**, see Table S2 and Figure S26, Supporting Information). For a fixed evaporation temperature of 50 °C, the resulting photonic pigments showed an increase in reflection wavelength with M_n_, with blue, green and red microparticles achieved with **CLEG2**–**4** under identical conditions (Figure S30, Supporting Information). However, when compared to samples of the same color (i.e. comparable S_p_, see Figure S14e), those prepared via temperature modulation typically demonstrate higher color saturation (Figure 1b, c compared to Figure S31a–f, Supporting Information). While for a given S_p_, the correlation length and pore size remain unchanged between the **CLEG1** series and those obtained with **CLEG2**–**4** (Figure S15b, c), this loss in color saturation can be attributed to a reduced correlation strength in terms of lower *ff* and much higher *w_r_*, as shown in Figure S15d and Figure S17b. As such, it suggests that producing pigments via modulation of the evaporation temperature is not only less laborious, but also results in a superior visual appearance compared to those prepared by varying the molecular weight of the constituent BBCP (Figure S32).

## Conclusion

In summary, we produced photonic pigments based on an inverse photonic glass architecture using a biocompatible BBCP. By analyzing the structural and optical properties, the correlation length, filling fraction, and pore size distribution have been determined to be key parameters for tuning the optical response of these materials. By combining this analysis with FDTD simulation of a reconstructed 3D foam structure, the relative contribution of these different parameters was deconvoluted, allowing for the origin of the different spectral peaks to be understood. In addition, analytical modeling was used to investigate the contributions of different scattering regimes. Using the predictions from both the simulation and analytical model we proposed a strategy to combine macromonomer blending with the evaporation temperature to improve the saturation and purity of the red colored pigment.

In conclusion, we reveal important insights into the origin of the optical response of inverse photonic glasses with low refractive index contrast and offer practical approaches to enhance their optical response, particularly at longer wavelengths. Furthermore, given the drive within the colorant industry to transition away from PET‐based polymer glitters and the use of unrenewable minerals (e.g. mica) in effect pigments, such biocompatible photonic BBCP microparticles expand the range of available approaches to sustainable photonic pigments beyond existing strategies based on colloidal self‐assembly.[^36^, ^37^]

## Conflict of interest

The authors declare no conflict of interest.

1

## Supporting information

As a service to our authors and readers, this journal provides supporting information supplied by the authors. Such materials are peer reviewed and may be re‐organized for online delivery, but are not copy‐edited or typeset. Technical support issues arising from supporting information (other than missing files) should be addressed to the authors.

Supporting Information

Supporting Information

## Data Availability

The data relating to this publication is openly available from the University of Cambridge data repository (https://doi.org/10.17863/CAM.85645).
